# Supplementary material for: Lipoteichoic Acid Accelerates Bone Healing by Enhancing Osteoblast Differentiation and Inhibiting Osteoclast Activation in a Mouse Model of Femoral Defects
Source: Int J Mol Sci. 2020 Aug 3;21(15):5550. doi: 10.3390/ijms21155550 (PMC7432397; doi:10.3390/ijms21155550)
Supplement: Supplementary file 1 [file ijms-21-05550-s001.pdf]

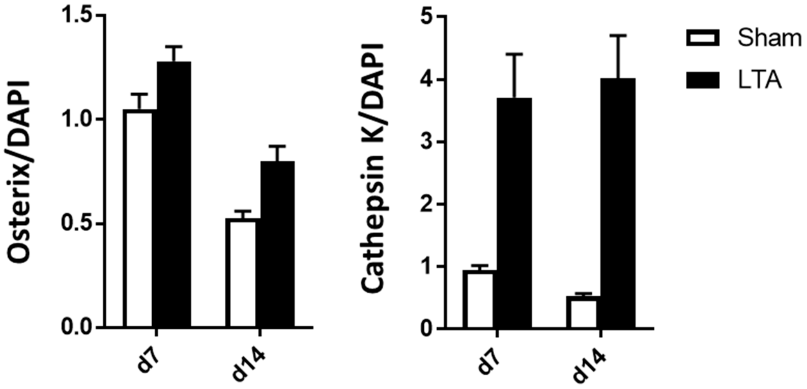

Supplementary figure 1. The intensities of osterix and cathepsin K signals relative to the area containing the bone tissue.
